# Exploratory studies to decide whether and how to proceed with full-scale evaluations of public health interventions: a systematic review of guidance

**DOI:** 10.1186/s40814-018-0290-8

**Published:** 2018-05-28

**Authors:** Britt Hallingberg, Ruth Turley, Jeremy Segrott, Daniel Wight, Peter Craig, Laurence Moore, Simon Murphy, Michael Robling, Sharon Anne Simpson, Graham Moore

**Affiliations:** 1 Centre for the Development and Evaluation of Complex Interventions for Public Health Improvement (DECIPHer), Cardiff University, Cardiff, Wales UK; 2 Centre for Trials Research, Cardiff University, Cardiff, Wales UK; 3 MRC/CSO Social and Public Health Sciences Unit, University of Glasgow, Glasgow, UK; 4 Specialist Unit for Review Evidence, Cardiff University, Cardiff, Wales UK

**Keywords:** Public health, Complex interventions, Exploratory studies, Research methods, Study design, Pilot study, Feasibility study

## Abstract

**Background:**

Evaluations of complex interventions in public health are frequently undermined by problems that can be identified before the effectiveness study stage. Exploratory studies, often termed pilot and feasibility studies, are a key step in assessing the feasibility and value of progressing to an effectiveness study. Such studies can provide vital information to support more robust evaluations, thereby reducing costs and minimising potential harms of the intervention. This systematic review forms the first phase of a wider project to address the need for stand-alone guidance for public health researchers on designing and conducting exploratory studies. The review objectives were to identify and examine existing recommendations concerning when such studies should be undertaken, questions they should answer, suitable methods, criteria for deciding whether to progress to an effectiveness study and appropriate reporting.

**Methods:**

We searched for published and unpublished guidance reported between January 2000 and November 2016 via bibliographic databases, websites, citation tracking and expert recommendations. Included papers were thematically synthesized.

**Results:**

The search retrieved 4095 unique records. Thirty papers were included, representing 25 unique sources of guidance/recommendations. Eight themes were identified: pre-requisites for conducting an exploratory study, nomenclature, guidance for intervention assessment, guidance surrounding any future evaluation study design, flexible versus fixed design, progression criteria to a future evaluation study, stakeholder involvement and reporting of exploratory studies. Exploratory studies were described as being concerned with the intervention content, the future evaluation design or both. However, the nomenclature and endorsed methods underpinning these aims were inconsistent across papers. There was little guidance on what should precede or follow an exploratory study and decision-making surrounding this.

**Conclusions:**

Existing recommendations are inconsistent concerning the aims, designs and conduct of exploratory studies, and guidance is lacking on the evidence needed to inform when to proceed to an effectiveness study.

**Trial registration:**

PROSPERO 2016, CRD42016047843

**Electronic supplementary material:**

The online version of this article (10.1186/s40814-018-0290-8) contains supplementary material, which is available to authorized users.

## Background

Improving public health and disrupting complex problems such as smoking, obesity and mental health requires complex, often multilevel, interventions. Such interventions are often costly and may cause unanticipated harms and therefore require evaluation using the most robust methods available. However, pressure to identify effective interventions can lead to premature commissioning of large effectiveness studies of poorly developed interventions, wasting finite research resources [1–3]. In the development of pharmaceutical drugs over 80% fail to reach ‘Phase III’ effectiveness trials, even after considerable investment [4]. With public health interventions, the historical tendency to rush to full evaluation has in some cases led to evaluation failures due to issues which could have been identified at an earlier stage, such as difficulties recruiting sufficient participants [5]. There is growing consensus that improving the effectiveness of public health interventions relies on attention to their design and feasibility [3, 6]. However, what constitutes good practice when deciding when a full evaluation is warranted, what uncertainties should be addressed to inform this decision and how, is unclear. This systematic review aims to synthesize existing sources of guidance for ‘exploratory studies’ which we broadly define as studies intended to generate evidence needed to decide whether and how to proceed with a full-scale effectiveness study. They do this by optimising or assessing the feasibility of the intervention and/or evaluation design that the effectiveness study would use. Hence, our definition includes studies variously referred to throughout the literature as ‘pilot studies’, ‘feasibility studies’ or ‘exploratory trials’. Our definition is consistent with previous work conducted by Eldridge et al. [7, 8], who define feasibility as an overarching concept [8] which assesses; ‘… whether the future trial can be done, should be done, and, if so, how’ (p. 2) [7]. However, our definition also includes exploratory studies to inform non-randomised evaluations, rather than a sole focus on trials.

The importance of thoroughly establishing the feasibility of intervention and evaluation plans prior to embarking on an expensive, fully powered evaluation was indicated in the Medical Research Council’s (MRC) framework for the development and evaluation of complex interventions to improve health [9, 10]. This has triggered shifts in the practice of researchers and funders toward seeking and granting funding for an ever growing number of studies to address feasibility issues. Such studies are however in themselves often expensive [11, 12]. While there is a compelling case for such studies, the extent to which this substantial investment in exploratory studies has to date improved the effectiveness and cost-effectiveness of evidence production remains to be firmly established. Where exploratory studies are conducted poorly, this investment may simply lead to expenditure of large amounts of additional public money, and several years’ delay in getting evidence into the hands of decision-makers, without necessarily increasing the likelihood that a future evaluation will provide useful evidence.

The 2000 MRC guidance used the term ‘exploratory trial’ for work conducted prior to a ‘definitive trial’, indicating that it should primarily address issues concerning the optimisation, acceptability and delivery of the intervention [13]. This included adaptation of the intervention, consideration of variants of the intervention, testing and refinement of delivery method or content, assessment of learning curves and implementation strategies and determining the counterfactual. Other possible purposes of exploratory trials included preliminary assessment of effect size in order to calculate the sample size for the main trial and other trial design parameters, including methods of recruitment, randomisation and follow-up. Updated MRC guidance in 2008 moved away from the sole focus on RCTs (randomised controlled trials) of its predecessor reflecting recognition that not all interventions can be tested using an RCT and that the next most robust methods may sometimes be the best available option [10, 14]. Guidance for exploratory studies prior to a full evaluation have, however, often been framed as relevant only where the main evaluation is to be an RCT [13, 15].

However, the goals of exploratory studies advocated by research funders have to date varied substantially. For instance, the National Institute for Health Research Evaluation Trials and Studies Coordinating Centre (NETSCC) definitions of feasibility and pilot studies do not include examination of intervention design, delivery or acceptability and do not suggest that modifications to the intervention prior to full-scale evaluation will arise from these phases. However, the NIHR (National Institute of Health Research) portfolio of funded studies indicates various uses of terms such as ‘feasibility trial’, ‘pilot trial’ and ‘exploratory trial’ to describe studies with similar aims, while it is rare for such studies not to include a focus on intervention parameters [16–18]. Within the research literature, there is considerable divergence over what exploratory studies should be called, what they should achieve, what they should entail, whether and how they should determine progression to future studies and how they should be reported [7, 8, 19–21].

This paper presents a systematic review of the existing recommendations and guidance on exploratory studies relevant to public health, conducted as the first stage of a project to develop new MRC guidance on exploratory studies. This review aims to produce a synthesis of current guidance/recommendations in relation to the definition, purpose and content of exploratory studies, and what is seen as ‘good’ and ‘bad’ practice as presented by the authors. It will provide an overview of key gaps and areas in which there is inconsistency within and between documents. The rationale for guidance and recommendations are presented, as well as the theoretical perspectives informing them. In particular, we examine how far the existing recommendations answer the following questions:

- When is it appropriate to conduct an exploratory study?
- What questions should such studies address?
- What are the key methodological considerations in answering these questions?
- What criteria should inform a decision on whether to progress to an effectiveness study?
- How should exploratory studies be reported?

## Methods

This review is reported in accordance with the PRISMA (Preferred Reporting Items for Systematic Reviews and Meta-Analyses) statement [22] as evidenced in the PRISMA checklist (see Additional file 1: Table S1). The review protocol is registered on PROSPERO (registration number: CRD42016047843; www.crd.york.ac.uk/prospero).

### Literature search

A comprehensive search (see Additional file 2: Appendix) was designed and completed during August to November 2016 to identify published and grey literature reported between January 2000 and November 2016 that contained guidance and recommendations on exploratory studies that could have potential relevance to public health. Bibliographic databases were CINAHL, Embase, MEDLINE, MEDLINE-In-process, PsycINFO, Web of Science and PubMed. Supplementary searches included key websites (see Additional file 2: Appendix) and forward and backward citation tracking of included papers, as well as contacting experts in the field. The first MRC guidance on developing and evaluating complex interventions in health was published in 2000; we therefore excluded guidance published before this year.

### Selection of included papers

Search results were exported into reference management software Endnote and clearly irrelevant or duplicate records removed by an information specialist. Eligibility criteria were applied to abstracts and potentially relevant full-text papers by two reviewers working independently in duplicate (BH, JS). Discrepancies were agreed by consensus or by a third reviewer if necessary. Full criteria are shown in Table 1. During screening of eligible studies, it became evident that determining whether or not guidance was applicable to public health was not always clear. The criteria in Table 1 were agreed by the team after a list of potentially eligible publications were identified.

**Table 1:** Eligibility criteria for selecting papers

	Eligibility criteria
Definition of exploratory study	• A study which aims to generate the evidence needed to decide whether and how to proceed with a full-scale effectiveness trial, or other study design and are labelled as exploratory/pilot/feasibility/phase II/proof of concept. Eligible publications may concern some or all of the design features of exploratory studies
Nature of guidance	• Guidance on the purpose, design, implementation or reporting of exploratory studies and/or • Other publication that reports ‘substantive information’ concerning the conduct of exploratory studies within public health—e.g. worked examples and methodological papers
Applicability to public health	• Public health audiences clearly among intended users of the guidance (authors are from Public Health departments, cites literature from public health journals, provides public health examples or uses the term ‘public health’ or variants of this, e.g. ‘prevention science’) or • Unspecific audience but of plausible relevance to public health (might, for example, include either an author from a public health research department or a citation to a public health journal)
Publication type/source	Book, book chapter, journal article, report or readily available doctoral thesis, funding organisation websites (UK and non-UK based)
Date and language restrictions	Publications reported since 2000 to date (November 2016), in any language.

### Quality assessment of included papers

Given the nature of publications included (expert guidance or methodological discussion papers) quality assessment was not applicable.

### Data extraction and thematic synthesis

A thematic synthesis of guidance within included documents was performed [23]. This involved the use of an a priori coding framework (based on the projects aims and objectives), developed by RT, JS and DW ([24], see Additional file 2: Appendix). Data were extracted using this schema in qualitative analytic software NVivo by one reviewer (BH). A 10% sample of coded papers was checked by a second reviewer (JS). Data were then conceptualised into final themes by agreement (BH, JS, DW, RT).

## Results

### Review statistics

**Fig. 1:**
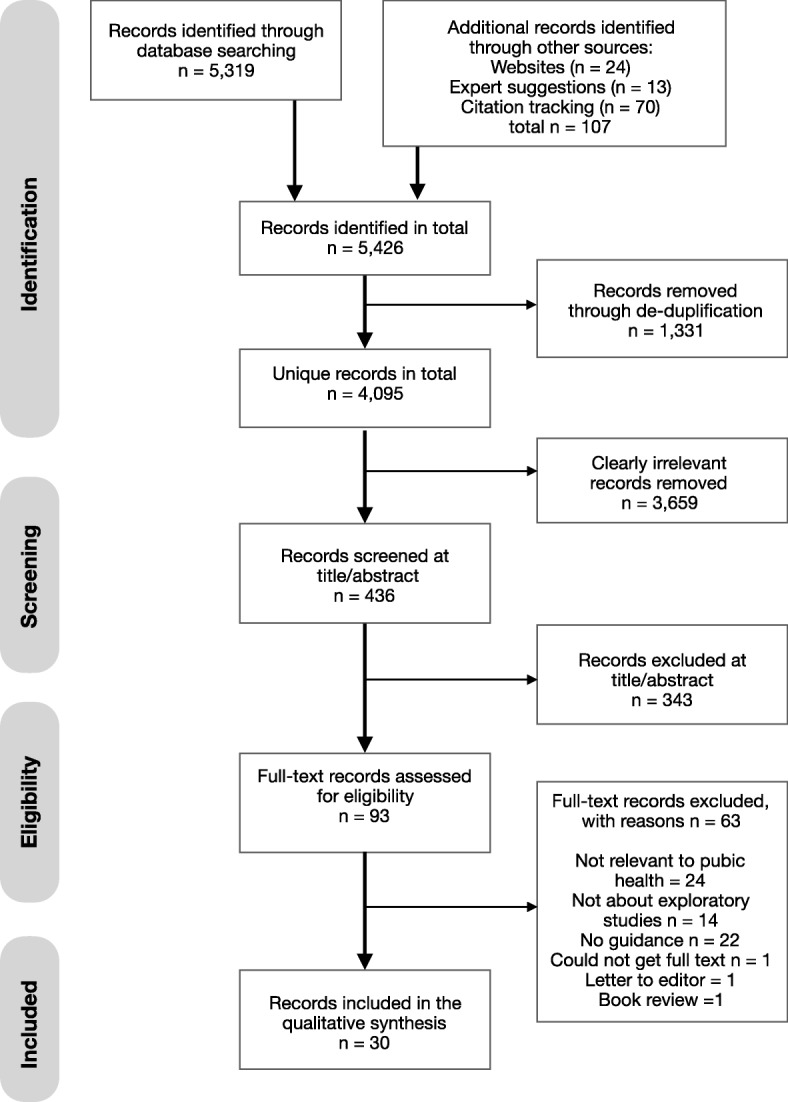
Flow diagram

Four thousand ninety-five unique records were identified of which 93 were reviewed in full text (see Fig. 1). In total, 30 documents were included in the systematic review representing 25 unique sets of guidance. Most sources of guidance did not explicitly identify an intended audience and guidance varied in its relevance to public health. Table 2 presents an overview of all sources of guidance included in the review with sources of guidance more or less relevant to public health identified as well as those which specifically applied to exploratory studies with a randomised design.

**Table 2:** Summary of included guidance

				Aims outlined and endorsed by authors^c^				
				Intervention			future evaluation	
First author(s)	Document type	Greater relevance to public health^a^	Nomenclature used	Refine	Implementation	Impact	Design feasibility	Parameter estimation
Arain et al. (2010) [19]	journal publication		pilot study^b^				x	
			feasibility study					x
Bowen et al. (2009) [39]	journal publication	✓	feasibility	x	x	x	x	
Campbell et al. (2000) [9]	journal publication	✓	phase II stage; exploratory trial	x	x			x
Cook et al. (2014) [35]	report to funder		pilot study					x
Craig et al. (2008) [10]	funder report	✓	feasibility/piloting stage; pilot study	x			x	x
Dixon-Woods et al. (2004) [23]	journal publication		exploratory clinical trial/phase II					
Eldridge, Chan et al. (2016) [7]	journal publication	✓	pilot trials^b^			x	x	x
			feasibility trials			x	x	x
Eldridge, Lancaster et al. (2016) [8]	journal publication	✓	pilot and feasibility studies^b^					
			-feasibility studies that are not pilot studies	x			x	
			-non-randomised pilot studies				x	
			-randomised pilot studies				x	
Eldridge, Costelloe et al. (2016) [41]	journal publication		pilot study		x	x	x	x
Evans et al. (2013) [28]	journal publication	✓	feasibility and piloting stage					
Feeley et al. (2009) [30]	journal publication		pilot study	x	x		x	x
Fletcher et al. (2016) [29]	journal publication	✓	pilot study^b^	x	x		x	
			feasibility study	x	x		x	
Hislop et al. (2014) [43]	journal publication		pilot study				x	x
Lancaster (2004) [36]	journal publication		pilot studies		x		x	x
Lancaster et al. (2015) [20]	journal publication		pilot and feasibility studies		x		x	x
Levati et al. (2016) [31]	journal publication	✓	pilot and feasibility studies	x				
Moffatt et al. (2006) [42]	journal publication (worked example)	✓	pilot study					
Möhler et al. (2012) [25]	journal publication		feasibility and piloting stage; pilot study		x			
Möhler et al. (2013) [26]	journal publication		feasibility and piloting stage; pilot study		x			
Möhler et al. (2015) [27]	journal publication		feasibility and piloting stage; pilot study		x			
Medical Research Council (2000) [13]	funder report	✓	phase II stage; exploratory trial	x	x	x	x	x
National Institute for Health Research, ‘Feasibility and Pilot studies’ (Accessed 14/10/16)	funder document		pilot study^b^			x		x
			feasibility study		x		x	x
National Institute for Health Research, ‘Progression rules for internal pilot studies for HTA trials’ (Accessed 14/10/16)	funder document		internal pilot studies				x	
National Institute for Health Research, ‘Glossary’ (14/10/16)	funder website		pilot study^b^			x	x	
			feasibility study				x	x
O'Cathain et al. (2015) [15]	journal publication	✓	feasibility study	x	x	x	x	
Shanyinde et al. (2011) [21]	journal publication		pilot / feasibility trials					
Strong et al. (2009) [40]	journal publication	✓	pilot intervention		x		x	
Taylor et al. (2015) [34]	book chapter	✓	pilot study^b^		x		x	
			feasibility study		x		x	
Westlund et al. (2016) [38]	journal publication		pilot study		x		x	
Wight et al. (2015) [6]	journal publication	✓	specific term not stated	x	x	x	x	x

^a^Guidance with greater relevance to public health included those where public health audiences was clearly among intended users of the guidance (authors are from Public Health departments, cites literature from public health journals, provides public health examples or uses the term ‘public health’ or variants of this, e.g. ‘prevention science’, ‘health improvement’). Guidance with less relevance was not specific about the intended audience but was of plausible relevance to public health (might, for example, include either an author from a public health research department or a citation to a public health journal).

^b^Authors make distinctions between the terms “pilot study” and “feasibility study”. ^c^ Aims of exploratory studies presented in the table map onto aims presented in themes 3 (*Guidance for intervention assessment*) and 4 (*Guidance surrounding the future evaluation design*)

### Findings from guidance

The included guidance reported a wide range of recommendations on the process of conducting and reporting exploratory studies. We categorised these into eight themes that capture: pre-requisites for conducting an exploratory study, nomenclature, guidance for intervention assessment, guidance surrounding the future evaluation study design, adaptive vs rigid designs, progression criteria for exploratory studies, stakeholder involvement and reporting.

### Narrative description of themes

#### Theme 1: pre-requisites for conducting an exploratory study

Where mentioned, pre-requisite activities included determining the evidence base, establishing the theoretical basis for the intervention, identifying the intervention components as well as modelling of the intervention in order to understand how intervention components interact and impact on final outcomes [9, 25–27]. These were often discussed within the context of the MRC’s intervention development-evaluation cycle [6, 9, 10, 13, 25–28]. Understanding how intervention components interact with various contextual settings [6, 27, 29] and identifying unintended harms [6, 29] as well as potential implementation issues [6, 9, 10, 30] were also highlighted. There was an absence of detail in judging when these above conditions were met sufficiently for moving onto an exploratory study.

#### Theme 2: nomenclature

A wide range of terms were used, sometimes interchangeably, to describe exploratory studies with the most common being pilot trial/study. Table 3 shows the frequency of the terms used in guidance including other terms endorsed.

**Table 3:** Frequency of nomenclature used

Nomenclature	Number of sources
Pilot trial/study	16
Feasibility trial/study	8
Feasibility and piloting stage	5
Pilot and/or feasibility trial/study	5
Phase II trial/study	3
Exploratory trial/study	3
Other terms (external pilot, feasibility studies but not pilot studies, non-randomised pilot studies, randomised feasibility studies, randomised pilot studies, exploratory pilot study, feasibility RCT, formative study, phase II stage, pilot RCT, process evaluation with a pilot trial, randomised feasibility trial, randomised pilot trial)	Terms presented once or twice across different sources of guidance.

Note: terms are not mutually exclusive

Different terminology did not appear to be consistently associated with specific study purposes (see theme 3), as illustrated in Table 2. ‘Pilot’ and ‘feasibility’ studies were sometimes used interchangeably [10, 20, 25–28, 31] while others made distinctions between the two according to design features or particular aims [7, 8, 19, 29, 32–34]. For example, some described pilot studies as a smaller version of a future RCT to run in miniature [7, 8, 19, 29, 32–34] and was sometimes associated with a randomised design [32, 34], but not always [7, 8]. In contrast, feasibility studies were used as an umbrella term by Eldridge et al. with pilot studies representing a subset of feasibility studies [7, 8]: ‘We suggest that researchers view feasibility as an overarching concept, with all studies done in preparation for a main study open to being called feasibility studies, and with pilot studies as a subset of feasibility studies.’ (p. 18) [8].

Feasibility studies could focus on particular intervention and trial design elements [29, 32] which may not include randomisation [32, 34]. Internal pilot studies were primarily viewed as part of the full trial [8, 32, 35–38] and are therefore not depicted under nomenclature in Table 3.

While no sources explicitly stated that an exploratory study should focus on one area and not the other, aims and associated methods of exploratory studies diverged into two separate themes. They pertained to either examining the intervention itself or the future evaluation design, and are detailed below in themes 3 and 4.

#### Theme 3: guidance for intervention assessment

Sources of guidance endorsed exploratory studies having formative purposes (i.e. refining the intervention and addressing uncertainties related to intervention implementation [13, 15, 29, 31, 39]) as well as summative goals (i.e. assessing the potential impact of an intervention or its promise [6, 13, 39]).

### Refining the intervention and underlying theory

Some guidance suggested that changes could be made within exploratory studies to refine the intervention and underlying theory [15, 29, 31] and adapt intervention content to a new setting [39]. However, guidance was not clear on what constituted minor vs. major changes and implications for progression criteria (see theme 6). When making changes to the intervention or underlying theory, some guidance recommended this take place during the course of the exploratory study (see theme 5). Others highlighted the role of using a multi-arm design to select the contents of the intervention before a full evaluation [13] and to assess potential mechanisms of multiple different interventions or intervention components [29]. Several sources highlighted the role of qualitative research in optimising or refining an intervention, particularly for understanding the components of the logic model [29] and surfacing hidden aspects of the intervention important for delivering outcomes [15].

### Intervention implementation

There was agreement across a wide range of guidance that exploratory studies could explore key uncertainties related to intervention implementation, such as acceptability, feasibility or practicality. Notably these terms were often ill-defined and used interchangeably. Acceptability was considered in terms of recipients’ reactions [7, 8, 29, 32, 39] while others were also attentive to feasibility from the perspective of intervention providers, deliverers and health professionals [6, 9, 29, 30, 34, 39]. Implementation, feasibility, fidelity and ‘practicality’ explored the likelihood of being able to deliver in practice what was intended [25–27, 30, 39]. These were sometimes referred to as aims within an embedded process evaluation that took place alongside an exploratory study, although the term process evaluation was never defined [7, 10, 15, 29, 40].

Qualitative research was encouraged for assessment of intervention acceptability [21] or for implementation (e.g. via non-participant observation [15]). Caution was recommended with regards to focus groups where there is a risk of masking divergent views [15]. Others recommended quantitative surveys to examine retention rates and reasons for dropout [7, 30]. Furthermore, several sources emphasised the importance of testing implementation in a range of contexts [15, 29, 39, 41]—especially in less socioeconomically advantaged groups, to examine the risk of widening health inequalities [29, 39].

One source of guidance considered whether randomisation was required for assessing intervention acceptability, believing this to be unnecessary but also suggesting it could ‘potentially depend on preference among interventions offered in the main trial’ ([21]; p. 9). Thus, issues of intervention acceptability, particularly within multi-arm trials, may relate to clinical equipoise and acceptability of randomisation procedures among participants [30].

### Appropriateness of assessing intervention impact

Several sources of guidance discussed the need to understand the impact of the intervention, including harms, benefits or unintended consequences [6, 7, 15, 29, 39]. Much of the guidance focused on statistical tests of effectiveness with disagreement on the soundness of this aim, although qualitative methods were also recommended [15, 42]. Some condemned statistically testing for effectiveness [7, 20, 29, 32, 41], as such studies are often underpowered, hence leading to imprecise and potentially misleading estimates of effect sizes [7, 20]. Others argued that an estimate of likely effect size could evidence the intervention was working as intended and not having serious unintended harms [6] and thus be used to calculate the power for the full trial [13]. Later guidance from the MRC is more ambiguous than earlier guidance, stating that estimates should be interpreted with caution, while simultaneously stating ‘safe’ assumptions of effect sizes as a pre-requisite before continuing to a full evaluation [10]. NIHR guidance, which distinguished between pilot and feasibility studies, supported the assessment of a primary outcome in pilot studies, although it is unclear whether this is suggesting that a pilot should involve an initial test of changes in the primary outcome, or simply that the primary outcome should be measured in the same way as it would be in a full evaluation. By contrast, for ‘feasibility studies’, it indicated that an aim may include designing an outcome measure to be used in a full evaluation.

Others made the case for identifying evidence of potential effectiveness, including use of interim or surrogate endpoints [7, 41], defined as ‘…variables on the causal pathway of what might eventually be the primary outcome in the future definitive RCT, or outcomes at early time points, in order to assess the potential for the intervention to affect likely outcomes in the future definitive RCT…’ [7] (p. 14).

Randomisation was implied as a design feature of exploratory studies when estimating an effect size estimate of the intervention as it maximised the likelihood that observed differences are due to intervention [9, 39], with guidance mostly written from a starting assumption that full evaluation will take the form of an RCT and guidance focused less on exploratory studies for quasi-experimental or other designs. For studies that aim to assess potential effectiveness using a surrogate or interim outcome, using a standard sample size calculation was recommended to ensure adequate power, although it was noted that this aim is rare in exploratory studies [7].

#### Theme 4: guidance surrounding the future evaluation design

Sources consistently advocated assessing the feasibility of study procedures or estimating parameters of the future evaluation. Recommendations are detailed below.

### Assessing feasibility of the future evaluation design

Assessing feasibility of future evaluation procedures was commonly recommended [6, 7, 10, 15, 30, 32–34, 37, 41] to avert problems that could undermine the conduct or acceptability of future evaluation [6, 15, 30]. A wide range of procedures were suggested as requiring assessments of feasibility including data collection [20, 30, 34, 36, 41], participant retention strategies [13], randomisation [7, 13, 20, 30, 34, 36, 38, 41], recruitment methods [13, 30, 32, 34, 35, 38, 41], running the full trial protocol [20, 30, 36], the willingness of participants to be randomised [30, 32] and issues of contamination [30]. There was disagreement concerning the appropriateness of assessing blinding in exploratory studies [7, 30, 34], with one source noting double blinding is difficult when participants are assisted in changing their behaviour; although assessing single blinding may be possible [30].

Qualitative [15, 30, 34], quantitative [34] and mixed methods [7] were endorsed for assessing these processes. Reflecting the tendency for guidance of exploratory studies to be limited to studies in preparation for RCTs, discussion of the role of randomisation at the exploratory study stage featured heavily in guidance. Randomisation within an exploratory study was considered necessary for examining feasibility of recruitment, consent to randomisation, retention, contamination or maintenance of blinding in the control and intervention groups, randomisation procedures and whether all the components of a protocol can work together, although randomisation was not deemed necessary to assess outcome burden and participant eligibility [21, 30, 34]. While there was consensus about what issues *could* be assessed through randomisation, sources disagreed on whether randomisation *should always* precede a future evaluation study, even if that future study is to be an RCT. Contention seemed to be linked to variation in nomenclature and associated aims. For example, some defined pilot study as a study run in miniature to test how all its components work together, thereby dictating a randomised design [32, 34]. Yet for feasibility studies, randomisation was only necessary if it reduced the uncertainties in estimating parameters for the future evaluation [32, 34]. Similarly, other guidance highlighted an exploratory study (irrespective of nomenclature) should address the main uncertainties, and thus may not depend on randomisation [8, 15].

### Estimating parameters of the future evaluation design

A number of sources recommended exploratory studies should inform the parameters of the future evaluation design. Areas for investigation included estimating sample sizes required for the future evaluation (e.g. measuring outcomes [32, 35]; power calculations [13]; derive effect size estimates [6, 7, 39]; estimating target differences [35, 43]; deciding what outcomes to measure and how [9, 20, 30, 36]; assessing quality of measures (e.g. for reliability/ validity/ feasibility/ sensitivity [7, 20, 30]; identification of control group [9, 13]; recruitment, consent and retention rates [10, 13, 20, 30, 32, 34, 36]; and information on the cost of the future evaluation design [9, 30, 36].

While qualitative methods were deemed useful for selecting outcomes and their suitable measures [15], most guidance concentrated on quantitative methods for estimating future evaluation sample sizes. This was contentious due to the potential to over- or under-estimate sample sizes required in a future evaluation due to the lack of precision of estimates from a small pilot [20, 30, 41]. Estimating sample sizes from effect size estimates in an exploratory study was nevertheless argued by some to be useful if there was scant literature and the exploratory study used the same design and outcome as the future evaluation [30, 39]. Cluster RCTs, which are common in public health interventions, were specifically earmarked as unsuitable for estimating parameters for sample size calculations (e.g. intra-cluster correlation coefficients) as well as recruitment and follow-up rates without additional information from other resources, because a large number of clusters and individual participants would be required [41]. Others referred to ‘rules of thumb’ when determining sample sizes in an exploratory study with numbers varying between 10 and 75 participants per trial arm in individually randomised studies [7, 30, 36]. Several also recommended the need to consider a desired meaningful difference in the health outcomes from a future evaluation and the appropriate sample size needed to detect this, rather than conducting sample size calculations using estimates of likely effect size from pilot data [30, 35, 38, 43].

A randomised design was deemed unnecessary for estimating costs or selecting outcomes, although was valued for estimating recruitment and retention rates for intervention and control groups [21, 34]. Where guidance indicated the estimation of an effect size appropriate to inform the sample size for a future evaluation, a randomised design was deemed necessary [9, 39].

#### Theme 5: flexible vs. fixed design

Sources stated that exploratory studies could employ a rigid or flexible design. With the latter, the design can change during the course of the study, which is useful for making changes to the intervention, as well as the future evaluation design [6, 13, 15, 31]. Here, qualitative data can be analysed as it is collected, shaping the exploratory study process, for instance sampling of subsequent data collection points [15], and clarifying implications for intervention effectiveness [31].

In contrast, fixed exploratory studies were encouraged when primarily investigating the future evaluation parameters and processes [13]. It may be that the nomenclature used in some guidance (e.g. pilot studies that are described as miniature versions of the evaluation) is suggesting a distinction between more flexible vs. more stringent designs. In some guidance, it was not mentioned whether changes should be made during the course of an exploratory study or afterwards, in order to get the best possible design for the future evaluation [6, 7, 21].

#### Theme 6: progression criteria to a future evaluation study

Little guidance was provided on what should be considered when formulating progression criteria for continuing onto a future evaluation study. Some focussed on the relevant uncertainties of feasibility [32, 39], while others highlight specific items concerning cost-effectiveness [10], refining causal hypotheses to be tested in a future evaluation [29] and meeting recruitment targets [20, 34]. As discussed in themes 3 and 4, statistically testing for effectiveness and using effect sizes for power calculations was cautioned by some, and so criteria based on effect sizes were not specified [38].

Greater discussion was devoted to how to weight evidence from an exploratory study that addressed multiple aims and used different methods. Some explicitly stated progression criteria should not be judged as strict thresholds but as guidelines using, for example, a traffic lights system with varying levels of acceptability [7, 41]. Others highlighted a realist approach, moving away from binary indicators to focusing on ‘what is feasible and acceptable for whom and under what circumstances’ [29]. In light of the difficulties surrounding interpretation of effect estimates, several sources recommended qualitative findings from exploratory studies should be more influential than quantitative findings [15, 38].

Interestingly, there was ambiguity regarding progression when exploratory findings indicated substantial changes to the intervention or evaluation design. Sources considering this issue suggested that if ‘extensive changes’ or ‘major modifications’ are made to either (note they did not specify what qualified as such), researchers should return to the exploratory [21, 30] or intervention development phases [15].

‘Alternatively, at the feasibility phase, researchers may identify fundamental problems with the intervention or trial conduct and return to the development phase rather than proceed to a full trial.’ (p. 1) [15].

As described previously, however, the threshold at which changes are determined to be ‘major’ remained ambiguous. While updated MRC guidance [10] moved to a more iterative model, accepting that movement back between feasibility/piloting and intervention development may sometimes be needed, there was no guidance on under what conditions movement between these two stages should take place.

#### Theme 7: stakeholder involvement

Several sources recommended a range of stakeholders (e.g. intervention providers, intervention recipients, public representatives as well as practitioners who might use the evidence produced by the full trial) be involved in the planning and running of the exploratory study to ensure exploratory studies reflect the realities of intervention setting [15, 28, 31, 32, 39, 40]. In particular, community-based participatory approaches were recommended [15, 39]. While many highlighted the value of stakeholders on Trial Steering Committees and other similar study groups [15, 28, 40], some warned about equipoise between researchers and stakeholders [15, 40] and also cautioned against researchers conflating stakeholder involvement with qualitative research [15].

‘Although patient and public representatives on research teams can provide helpful feedback on the intervention, this does not constitute qualitative research and may not result in sufficiently robust data to inform the appropriate development of the intervention.’ (p. 8) [15].

#### Theme 8: reporting of exploratory studies

Detailed recommendations for reporting exploratory studies were recently provided in new Consolidated Standards of Reporting Trials (CONSORT) guidance by Eldridge et al. [7]. In addition to this, recurrent points were brought up by other sources of guidance. Most notably, it was recommended exploratory studies be published in peer-reviewed journals as this can provide useful information to other researchers on what has been done, what did not work and what might be most appropriate [15, 30]. An exploratory study may also result in multiple publications, but should provide reference to other work carried out in the same exploratory study [7, 15]. Several sources of guidance also highlight that exploratory studies should be appropriately labelled in the title/abstract to enable easy identification; however, the nomenclature suggested varied depending on guidance [7, 8, 15].

## Discussion

While exploratory studies—carried out to inform decisions about whether and how to proceed with an effectiveness study [7, 8]—are increasingly recognised as important in the efficient evaluation of complex public health interventions, our findings suggest that this area remains in need of consistent standards to inform practice. At present, there are multiple definitions of exploratory studies, a lack of consensus on a number of key issues, and a paucity of detailed guidance on how to approach the main uncertainties such studies aim to address prior to proceeding to a full evaluation.

Existing guidance commonly focuses almost exclusively on testing methodological parameters [33], such as recruitment and retention, although in practice, it is unusual for such studies not to also focus on the feasibility of the intervention itself. Where intervention feasibility is discussed, there is limited guidance on when an intervention is ‘ready’ for an exploratory study and a lack of demarcation between intervention development and pre-evaluation work to understand feasibility. Some guidance recognised that an intervention continues to develop throughout an exploratory study, with distinctions made between ‘optimisation/refinement’ (i.e. minor refinements to the intervention) vs. ‘major changes’. However, the point at which changes become so substantial that movement back toward intervention development rather than forward to a full evaluation remains ambiguous. Consistent with past reviews which adopted a narrower focus on studies with randomised designs [21] or in preparation for a randomised trial [8, 36] and limited searches of guidance in medical journals [19, 36], terms to describe exploratory studies were inconsistent, with a distinction sometimes made between pilot and feasibility studies, though with others using these terms interchangeably.

The review identifies a number of key areas of disagreement or limited guidance in regards to the critical aims of exploratory studies and addressing uncertainties which might undermine a future evaluation, and how these aims should be achieved. There was much disagreement for example on whether exploratory studies should include a preliminary assessment of intervention effects to inform decisions on progression to a full evaluation, and the appropriateness of using estimates of effect from underpowered data (from non-representative samples and a study based on a not fully optimised version of the intervention) to power a future evaluation study. Most guidance focused purely on studies in preparation for RCTs; nevertheless, guidance varied on whether randomisation was a necessary feature of the exploratory study, even where a future evaluation study was an RCT. Guidance was often difficult to assess regarding its applicability to public health research, with many sources focusing on literature and practice primarily from clinical research, and limited consideration of the transferability of these problems and proposed solutions to complex social interventions, such as those in public health. Progression criteria were highlighted as important by some as a means of preventing biased post hoc cases for continuation. However, there was a lack of guidance on how to devise progression criteria and processes for assessing whether these had been sufficiently met. Where they had not been met, there was a lack of guidance on how to decide whether the exploratory study had generated sufficient insight about uncertainties that the expense of a further feasibility study would not be justified prior to large-scale evaluation.

Although our review included a broad focus on guidance of exploratory studies from published and grey literature and moved beyond a focus on studies conducted in preparation for an RCT specifically, a number of limitations should be noted. Guidance from other areas of social intervention research where challenges may be similar to those in public health (e.g. education, social work and business) may not have been captured by our search strategy. We found few worked examples of exploratory studies in public health that provided substantial information from learned experience and practice. Hence, the review drew largely on recommendations from funding organisations, or relatively abstract guidance from teams of researchers, with fewer clear examples of how these recommendations are grounded in experience from the conduct of such studies. As such, it should be acknowledged that these documents represent one element within a complex system of research production and may not necessarily fully reflect what is taking place in the conduct of exploratory studies. Finally, treating sources of guidance as independent from each other does not reflect how some recommendations developed over time (see for example [7, 8, 20, 36, 41]).

## Conclusion

There is inconsistent guidance, and for some key issues a lack of guidance, for exploratory studies of complex public health interventions. As this lack of guidance for researchers in public health continues, the implications and consequences could be far reaching. It is unclear how researchers use existing guidance to shape decision-making in the conduct of exploratory studies, and in doing so, how they adjudicate between various conflicting perspectives. This systematic review has aimed largely to identify areas of agreement and disagreement as a starting point in bringing order to this somewhat chaotic field of work. Following this systematic review, our next step is to conduct an audit of published public health exploratory studies in peer-reviewed journals, to assess current practice and how this reflects the reviewed guidance. As part of a wider study, funded by the MRC/NIHR Methodology Research Programme to develop GUidance for Exploratory STudies of complex public health interventions (GUEST; Moore L, et al. Exploratory studies to inform full scale evaluations of complex public health interventions: the need for guidance, submitted), the review has informed a Delphi survey of researchers, funders and publishers of public health research. In turn, this will contribute to a consensus meeting which aims to reach greater unanimity on the aims of exploratory studies, and how these can most efficiently address uncertainties which may undermine a full-scale evaluation.

## Additional files

Additional file 1: **Table S1.** PRISMA checklist. (DOC 62 kb)

Additional file 2: **Appendix 1.** Search strategies and websites. **Appendix 2.** Coding framework. (DOCX 28 kb)

## Abbreviations

CONSORT: Consolidated Standards of Reporting Trials
GUEST: GUidance for Exploratory STudies of complex public health interventions
MRC: Medical Research Council
NETSCC: National Institute of Health Research Evaluation Trials and Studies Coordinating Centre
NIHR: National Institute for Health Research
PRISMA: Preferred Reporting Items for Systematic Reviews and Meta-Analyses
RCT: Randomised controlled trial
