# Supplementary material for: Exploratory studies to decide whether and how to proceed with full-scale evaluations of public health interventions: a systematic review of guidance
Source: Pilot Feasibility Stud. 2018 May 28;4:104. doi: 10.1186/s40814-018-0290-8 (PMC5971430; doi:10.1186/s40814-018-0290-8)
Supplement: Supplementary file 2 — Appendix 1. Search strategies and websites. Appendix 2. Coding framework. (DOCX 28 kb) [file 40814_2018_290_MOESM2_ESM.docx]

**Appendix 1: Search strategies and websites**

**Website sources**

Medical Research Council

National Institute Health Research

National Institute for Health and Clinical Excellence (NICE)

CONSORT

Equator Network

World Health Organisation

Agency for Healthcare Research and Quality

NHS Research Design Service

Wellcome Trust

UK Society for Behavioural Medicine

Society for Social Medicine

Society of Behavioural Medicine

American Institutes for Research

Canadian Institutes of Health Research

Substance Abuse and Mental Health Services Administration (SAMSHA)

Centre for Disease Control and Prevention (CDC)

National Institute on Alcohol Abuse and Alcoholism (NIAAA)

National Institute on Drug Abuse (NIDA)

National Institute of Mental Health (NIMH)

Economic and Social Research Council (ESRC)

**Bibliographic database search strategies**

Cinahl

S1 TI ( (recommend*) N5 (pilot or feasibility or "validation study" or "validation trial" or exploratory or “proof of principle” or "preliminary trial*") )

S2 TI ( (("good practice") N5 (pilot or feasibility or "validation study" or "validation trial" or exploratory or "proof of principle" or "preliminary trial*")) ) OR AB ( (("good practice") N5 (pilot or feasibility or "validation study" or "validation trial" or exploratory or "proof of principle" or "preliminary trial*")) )

S3 TI ( (("best practice") N5 (pilot or feasibility or "validation study" or "validation trial" or exploratory or "proof of principle" or "preliminary trial*")) ) OR AB ( (("best practice") N5 (pilot or feasibility or "validation study" or "validation trial" or exploratory or "proof of principle" or "preliminary trial*")) )

S4 TI ((guidance) N5 (pilot or feasibility or "validation study" or "validation trial" or exploratory or "proof of principle" or "preliminary trial*"))

S5 TI ((guidelines) N5 (pilot or feasibility or "validation study" or "validation trial" or exploratory or "proof of principle" or "preliminary trial*"))

S6 TI ( ((advice) N5 (pilot or feasibility or "validation study" or "validation trial" or exploratory or "proof of principle" or "preliminary trial*")) ) OR AB ( ((advice) N5 (pilot or feasibility or "validation study" or "validation trial" or exploratory or "proof of principle" or "preliminary trial*")) )

S7 TI ( ((advised) N5 (pilot or feasibility or "validation study" or "validation trial" or exploratory or "proof of principle" or "preliminary trial*")) ) OR AB ( ((advised) N5 (pilot or feasibility or "validation study" or "validation trial" or exploratory or "proof of principle" or "preliminary trial*")) )

S8 TI ( ((tutorial*) N5 (pilot or feasibility or "validation study" or "validation trial" or exploratory or "proof of principle" or "preliminary trial*")) ) OR AB ( ((tutorial*) N5 (pilot or feasibility or "validation study" or "validation trial" or exploratory or "proof of principle" or "preliminary trial*")) )

S9 TI ( (("method* development") N5 (pilot or feasibility or "validation study" or "validation trial" or exploratory or "proof of principle" or "preliminary trial*")) ) OR AB ( (("method* development") N5 (pilot or feasibility or "validation study" or "validation trial" or exploratory or "proof of principle" or "preliminary trial*")) )

S10 TI ( (("method* insight") N5 (pilot or feasibility or "validation study" or "validation trial" or exploratory or "proof of principle" or "preliminary trial*")) ) OR AB ( (("method* insight") N5 (pilot or feasibility or "validation study" or "validation trial" or exploratory or "proof of principle" or "preliminary trial*")) )

S11 TI ( (("method* research") N5 (pilot or feasibility or "validation study" or "validation trial" or exploratory or "proof of principle" or "preliminary trial*")) ) OR AB ( (("method* research") N5 (pilot or feasibility or "validation study" or "validation trial" or exploratory or "proof of principle" or "preliminary trial*")) )

S12 TI ( ((rigor or rigour) N5 (pilot or feasibility or "validation study" or "validation trial" or exploratory or "proof of principle" or "preliminary trial*")) ) OR AB ( ((rigor or rigour) N5 (pilot or feasibility or "validation study" or "validation trial" or exploratory or "proof of principle" or "preliminary trial*")) )

S13 S1 OR S2 OR S3 OR S4 OR S5 OR S6 OR S7 OR S8 OR S9 OR S10 OR S11 OR S12

Embase

1. feasibility studies/

2. evaluation studies as topic/ and (pilot or feasibility or validation or small scale or exploratory or preliminary or "proof of principal" or MRC).ti,ab.

3. *validation study/

4. (phase adj (II or "2" or two)).ti,ab.

5. clinical trials, phase II as topic/

6. pilot projects/

7. ((pilot or feasibility or validation or small scale) adj2 (study or studies or trial or trial*1 or RCT*1 or randomi#ed)).tw.

8. ((exploratory or "proof of principle") adj2 (study or studies or trial or trials or RCT)).tw.

9. preliminary trial*1.ti,ab.

10. exploratory research.ti,ab.

11. or/1-10

12. ((guidance or guidelines) adj3 (method* or conduct or design or approach)).ti,ab.

13. ((Recommend$ or standards or good practice or best practice) adj2 (method* or conduct* or reporting or design or approach)).ti,ab.

14. ((impact or effect*) adj3 (guidance or guidelines or recommend* or standards or good practice or best practice)).ti,ab.

15. (progression criteria adj3 (guidance or guidelines or recommend* or standards or good practice or best practice)).ti,ab.

16. or/12-15

17. 11 and 16

18. (method* adj2 (discussion or indepth or insight* or consideration* or implication*)).ti,ab.

19. ((method or methodology) adj (challenge* or lesson* or worked)).ti.

20. exemplar.ti,ab.

21. methodological.ti.

22. (method* adj2 (develop* or standard* or example)).ti,ab.

23. (methodology research or methodological research).ti,ab.

24. (lesson$1 learned adj5 (method$ or conduct$)).ti,ab.

25. or/18-24

26. exp Attitude to Health/

27. health education/

28. public health/ or (prevention science or prevention research).ti,ab.

29. risk reduction behavior/

30. harm reduction/ or health behavior/

31. exp health promotion/

32. ((Behavio?r* or attitude* or habit*1 or lifestyle) adj3 (change$ or modif* or intervention* or adapt*)).ti,ab.

33. ((smoking or tobacco or alcohol misuse or binge drinking or drug misuse or substance misuse or parenting) adj3 (improv$ or reduc$ or prevent$ or chang$ or intervention)).ti,ab.

34. ((health or wellbeing) adj2 (improv* or intervention)).ti,ab.

35. (Health$ adj3 (behavio?r$ or lifestyle$)).tw.

36. ((nutrition or diet or physical activity or physical inactivity or sedentary or obesity or dietary or healthy diet* or sexual behavio#r or risk$ behavio#r or mental health or emotional wellbeing or suicid* or self-harm*) adj3 (improv$ or reduc$ or prevent$ or chang$)).ti,ab.

37. ((workplace* or school*1 or education settings or communit* or famil* or community-based or prison*1) adj3 setting*).ti,ab.

38. (public health adj3 (complex or MRC or medical research council or complex or intervention*)).ti,ab.

39. (complex intervention* or complex public health intervention*).ti,ab.

40. or/26-39

41. 11 and 25 and 40

42. (((Recommend$ or "good practice" or "best practice" or guidance or guidelines or advice or advised or tutorial* or method$ development or method$ insight or method$ research) adj5 (pilot or feasibility or exploratory or "proof of principle" or preliminary trial*1)) and (stud$ or research$)).ti,ab.

43. 17 or 41 or 42

44. limit 43 to yr="2000 - 2016"

Medline & Medline in Process

1 feasibility studies/

2 evaluation studies as topic/ and (pilot or feasibility or validation or small scale or exploratory or preliminary or "proof of principal" or MRC).ti,ab.

3 validation studies/

4 (phase adj (II or "2" or two)).ti,ab.

5 clinical trials, phase II as topic/

6 pilot projects/

7 ((pilot or feasibility or validation or small scale) adj2 (study or studies or trial or trial*1 or RCT*1 or randomi#ed)).tw.

8 ((exploratory or "proof of principle") adj2 (study or studies or trial or trials or RCT)).tw.

9 preliminary trial*1.ti,ab.

10 exploratory research.ti,ab.

11 or/1-10

12 ((guidance or guidelines) adj3 (method* or conduct or design or approach)).ti,ab.

13 ((Recommend$ or standards or good practice or best practice) adj2 (method* or conduct* or reporting or design or approach)).ti,ab.

14 ((impact or effect*) adj3 (guidance or guidelines or recommend* or standards or good practice or best practice)).ti,ab.

15 (progression criteria adj3 (guidance or guidelines or recommend* or standards or good practice or best practice)).ti,ab.

16 or/12-15

17 11 and 16

18 (method* adj2 (discussion or indepth or insight* or consideration* or implication*)).ti,ab.

19 ((method or methodology) adj (challenge* or lesson* or worked)).ti.

20 exemplar.ti,ab.

21 methodological.ti.

22 (method* adj2 (develop* or standard* or example)).ti,ab.

23 (methodology research or methodological research).ti,ab.

24 (lesson$1 learned adj5 (method$ or conduct$)).ti,ab.

25 or/18-24

26 exp Attitude to Health/

27 health education/

28 public health/ OR (prevention science or prevention research).ti,ab

29 risk reduction behavior/

30 harm reduction/ or health behavior/

31 exp health promotion/

32 ((Behavio?r* or attitude* or habit*1 or lifestyle) adj3 (change$ or modif* or intervention* or adapt*)).ti,ab.

33 ((smoking or tobacco or alcohol misuse or binge drinking or drug misuse or substance misuse or parenting) adj3 (improv$ or reduc$ or prevent$ or chang$ or intervention)).ti,ab.

34 ((health or wellbeing) adj2 (improv* or intervention).ti,ab.

35 (Health$ adj3 (behavio?r$ or lifestyle$)).tw.

36 ((nutrition or diet or physical activity or physical inactivity or sedentary or obesity or dietary or healthy diet* or sexual behavio#r or risk$ behavio#r or mental health or emotional wellbeing or suicid* or self-harm*) adj3 (improv$ or reduc$ or prevent$ or chang$)).ti,ab.

37 ((workplace* or school*1 or education settings or communit* or famil* or community-based or prison*1) adj3 setting*).ti,ab.

38 (public health adj3 (complex or MRC or medical research council or complex or intervention*)).ti,ab.

39 (complex intervention* or complex public health intervention).ti,ab.

40 or/26-39

41 11 and 25 and 40

42 ((Recommend$ or "good practice" or "best practice" or guidance or guidelines or advice or advised or tutorial* or method$ development or method$ insight or method$ research) adj5 (pilot or feasibility or validation study or validation trial or exploratory or "proof of principle" or preliminary trial*1)).ti,ab.

43 17 or 41 or 42

44 limit 43 to yr="2000 -Current"

Psycinfo

1. feasibility studies.ti,ab.

2. (phase adj (II or "2" or two)).ti,ab.

3. ((pilot or feasibility or validation or small scale) adj2 (study or studies or trial or trial*1 or RCT*1 or randomi#ed)).tw.

4. ((exploratory or "proof of principle") adj2 (study or studies or trial or trials or RCT)).tw.

5. preliminary trial*1.ti,ab.

6. exploratory research.ti,ab.

7. or/1-6

8. ((guidance or guidelines) adj3 (method* or conduct or design or approach)).ti,ab.

9. ((Recommend$ or standards or good practice or best practice) adj2 (method* or conduct* or reporting or design or approach)).ti,ab.

10. ((impact or effect*) adj3 (guidance or guidelines or recommend* or standards or good practice or best practice)).ti,ab.

11. (progression criteria adj3 (guidance or guidelines or recommend* or standards or good practice or best practice)).ti,ab.

12. or/8-11

13. 7 and 12

14. (method* adj2 (discussion or indepth or insight* or consideration* or implication*)).ti,ab.

15. ((method or methodology) adj (challenge* or lesson* or worked)).ti.

16. exemplar.ti,ab.

17. methodological.ti.

18. (method* adj2 (develop* or standard* or example)).ti,ab.

19. (methodology research or methodological research).ti,ab.

20. (lesson$1 learned adj5 (method$ or conduct$)).ti,ab.

21. or/14-20

22. exp Attitude to Health/

23. health education/

24. public health/ or (prevention science or prevention research).ti,ab.

25. risk reduction/

26. harm reduction/ or health behavior/

27. exp health promotion/

28. ((Behavio?r* or attitude* or habit*1 or lifestyle) adj3 (change$ or modif* or intervention* or adapt*)).ti,ab.

29. ((smoking or tobacco or alcohol misuse or binge drinking or drug misuse or substance misuse or parenting) adj3 (improv$ or reduc$ or prevent$ or chang$ or intervention)).ti,ab.

30. (((health or wellbeing) adj2 (improv* or intervention).ti,ab.

31. (Health$ adj3 (behavio?r$ or lifestyle$)).tw.

32. ((nutrition or diet or physical activity or physical inactivity or sedentary or obesity or dietary or healthy diet* or sexual behavio#r or risk$ behavio#r or mental health or emotional wellbeing or suicid* or self-harm*) adj3 (improv$ or reduc$ or prevent$ or chang$)).ti,ab.

33. ((workplace* or school*1 or education settings or communit* or famil* or community-based or prison*1) adj3 setting*).ti,ab.

34. (public health adj3 (complex or MRC or medical research council or complex or intervention*)).ti,ab.

35. (complex intervention* or complex public health intervention*).ti,ab.

36. or/22-35

37. 7 and 21 and 36

38. ((Recommend$ or "good practice" or "best practice" or guidance or guidelines or advice or advised or tutorial* or method$ development or method$ insight or method$ research) adj5 (pilot or feasibility or validation study or validation trial or exploratory or "proof of principle" or preliminary trial*1)).ti,ab.

39. 13 or 37 or 38

40. limit 39 to yr="2000 - 2016"

Pubmed

((((((recommend*[Title] OR rigour[Title/Abstract] OR rigor[Title/Abstract] OR “good practice”[Title/Abstract] OR “best practice”[Title/Abstract] OR guidance[Title] OR guidelines[Title] OR advice[Title/Abstract] OR advised[Title/Abstract] OR tutorial*[Title/Abstract] OR "method* development"[Title/Abstract] OR "method* insight"[Title/Abstract] OR "method* research"[Title/Abstract])) AND (pilot[Title/Abstract] OR feasibility[Title/Abstract] OR "validation study"[Title/Abstract] OR "validation trial"[Title/Abstract] OR exploratory[Title/Abstract] OR “proof of principle”[Title/Abstract] OR "preliminary trial*"[Title/Abstract])) AND pubstatusaheadofprint)))

Web of Science

*Indexes=SCI-EXPANDED, SSCI, CPCI-S, CPCI-SSH Timespan=2000-2016*

# 1 TI=((recommend*) NEAR/5 (pilot or feasibility or "validation study" or "validation trial" or exploratory or "proof of principle" or "preliminary trial*"))

# 2 TS=(("good practice") NEAR/5 (pilot or feasibility or "validation study" or "validation trial" or exploratory or "proof of principle" or "preliminary trial*"))

# 3 TS=(("best practice") NEAR/5 (pilot or feasibility or "validation study" or "validation trial" or exploratory or "proof of principle" or "preliminary trial*"))

# 4 TI=((guidance) NEAR/5 (pilot or feasibility or "validation study" or "validation trial" or exploratory or "proof of principle" or "preliminary trial*"))

# 5 TI=((guidelines) NEAR/5 (pilot or feasibility or "validation study" or "validation trial" or exploratory or "proof of principle" or "preliminary trial*"))

# 6 TS=((advice) NEAR/5 (pilot or feasibility or "validation study" or "validation trial" or exploratory or "proof of principle" or "preliminary trial*"))

# 7 TS=((advised) NEAR/5 (pilot or feasibility or "validation study" or "validation trial" or exploratory or "proof of principle" or "preliminary trial*"))

# 8 TS=((tutorial*) NEAR/5 (pilot or feasibility or "validation study" or "validation trial" or exploratory or "proof of principle" or "preliminary trial*"))

# 9 TS=(("method* development") NEAR/5 (pilot or feasibility or "validation study" or "validation trial" or exploratory or "proof of principle" or "preliminary trial*"))

# 10 TS=(("method* insight") NEAR/5 (pilot or feasibility or "validation study" or "validation trial" or exploratory or "proof of principle" or "preliminary trial*"))

# 11 TS=(("method* research") NEAR/5 (pilot or feasibility or "validation study" or "validation trial" or exploratory or "proof of principle" or "preliminary trial*"))

# 12 TS=((rigor or rigour) NEAR/5 (pilot or feasibility or "validation study" or "validation trial" or exploratory or "proof of principle" or "preliminary trial*"))

# 13 #12 OR #11 OR #10 OR #9 OR #8 OR #7 OR #6 OR #5 OR #4 OR #3 OR #2 OR #1

Appendix 2: Coding framework

| **Name** | **notes** |
| --- | --- |
| Nomenclature | Nomenclature used and definition |
| Exploratory trial / study |  |
| Feasibility trial / study |  |
| Phase II trial / study |  |
| Pilot trial / study |  |
| Other trial / study | Ex: intervention development, acceptability trial/study |
|  |  |
| Circumstances requiring exploratory study |  |
| Pre-requisites | Pre-requisites for conducting an exploratory study. Work needed before appropriateness of conducting an exploratory study can be determined. Evidence needed to justify conducting exploratory study (e.g. intervention logic model, information on initial acceptability of intervention). |
| Author’s aims | Aims of an exploratory study endorsed by the author of the guidance |
| Test feasibility of trial procedures | Test procedures and processes of trial (randomisation process, recruitment, data collection etc.) |
| Estimate key parameters for trial | Estimate key parameters needed to design main study (information on outcome measure to estimate sample size and target difference, number of eligible participants, response rates/retention, timing of data collection and analyses, economic evaluation etc.) |
| Feasibility of effectiveness trial | Feasibility of conducting an effectiveness trial |
| Explore uncertainties of intervention | Identify and address problems of intervention |
| Assess acceptability of intervention | Investigation of acceptability of intervention for participants, those delivering intervention etc. |
| Transferability of intervention between settings | Cultural adaptation of intervention and transferring intervention to other settings |
| Intervention optimization | Intervention modification and refinement. Have model but developing intervention (development/design on intervention measures). |
| Understand mechanisms of intervention | Provides opportunity to understand interactions amongst inputs and outputs, test logic model |
| Feasibility of intervention implementation | Feasibility of intervention implementation, Identifying/solving implementation problems |
| Evaluation of intervention effectiveness | Use of pilot to test intervention outcomes (to justify grant award, identify potential effectiveness etc.) |
| Explore uncertainties of evaluation | Identify and address problems of evaluation |
| Economic Evaluation | Use of pilot economic evaluation to inform design of full trial |
|  |  |
| Methodological considerations |  |
| Design | Guidance on the design of exploratory studies |
| Intervention implementation | Full or partial implementation of intended interventions, Do you implement the intervention and to what extent? |
| Process evaluation | Inclusion of process evaluation |
| Randomisation |  |
| Appropriateness of randomisation | Is randomisation appropriate in the exploratory study (particularly if planned in the full trial)? |
| Rationale for randomisation | (e.g. test feasibility, necessary for evaluation of effectiveness during exploratory study). |
| Setting-specific issues | How to implement in practice, changes to protocol etc. |
| Methods |  |
| Mixed methods | Combining both quantitative and qualitative methods in exploratory studies |
| Qualitative methods | Use of qualitative methods in exploratory studies |
| Quantitative methods | Use of quantitative methods in exploratory studies |
| Sample |  |
| Calculation of effect and sample size for an effectiveness study | How to calculate effect and sample size for effectiveness study. |
| Heterogeneity of sample | Heterogeneity of sample in feasibility study and potential for bias. |
| Theory Frameworks | Theoretical perspectives/methodological frameworks referred to by guidance. |
| Formal guidance | ex: MRC, NIHR guidance, CONSORT |
| User and public involvement | Guidance on including a participatory approach in an exploratory study |
| Analysis | Guidance on how to analyse data from exploratory studies (mixing quantitative & qualitative results) |
| Reporting | Guidance on reporting exploratory study (writing up aims etc) |
|  |  |
| Criteria for progressing to an effectiveness trial |  |
| Formulation of criteria | Which progression criteria should be included and how should they be formulated? |
| Assessment of criteria | How should progression criteria be assessed individually and combined to provide an overall decision on whether to proceed to an effectiveness trial? |
| Action following assessment | Next steps to be taken if decision is not to proceed with full trial. |
|  |  |
| Application of Guidance | Audience/area of research guidance is directed at or should be applied to. |
